# Recognizing Presentations of Pemphigoid Gestationis: A Case Study

**DOI:** 10.1155/2014/415163

**Published:** 2014-11-18

**Authors:** Sadie Henry

**Affiliations:** Naval Medical Center Portsmouth, 620 John Paul Jones Circle, Portsmouth, VA 23708, USA

## Abstract

*Introduction*. Pemphigoid gestationis (PG) is an autoimmune blistering disease that occurs in approximately 1 in 50,000 pregnancies. Failing to recognize PG may lead to inadequate maternal treatment and possible neonatal complications. *Case Report*. At 18 weeks of gestation, a 36-year-old otherwise healthy Caucasian G4P1 presented with pruritic papules on her anterior thighs, initially treated with topical steroids. At 31 weeks of gestation, she was switched to oral steroids after her rash and pruritus worsened. The patient had an uncomplicated SVD of a healthy female infant at 37 weeks of gestation and was immediately tapered off steroid treatment, resulting in a severe postpartum flare of her disease. *Discussion*. This case was similar to reported cases of pruritic urticarial papules followed by blisters; however, this patient had palm, sole, and mucous membrane involvement, which is rare. Biopsy for direct immunofluorescence or ELISA is the preferred test for diagnosis. Previous case reports describe severe postdelivery flares that require higher steroid doses. Obstetrical providers need to be familiar with this disease although it is rare, as this condition can be easily confused with other dermatoses of pregnancy. Adequate treatment is imperative for the physical and psychological well-being of the mother and infant.

## 1. Introduction

Pemphigoid gestationis (PG), formally herpes gestationis (HG), is an autoimmune vesiculobullous skin disease that occurs in approximately 1 in 50,000 pregnancies [1]. It has also been associated with trophoblastic tumors such as a hydatidiform mole or choriocarcinoma. Diagnosis is made based on the presence of subepidermal vesicles and linear deposition of C3 along the basement membrane of the skin [2]. Abnormal expression of major histone complex (MHC) class II molecules in the placenta triggers an immunologic event, which is followed by cross-reactivity with an antigen present in the skin [3]. This leads to activation of the inflammatory cascade, including the classical complement pathway causing the characteristic vesicles and bulla [4]. Not recognizing pemphigoid gestationis may lead to inadequate maternal treatment and possible preterm birth and neonatal complications.

The purpose of the case report is to make clinicians more aware of PG, to include it in their differential diagnosis, and to know general management guidelines. This report describes an uncommon distribution of this uncommon disease and provides an opportunity to review and correct any actions that may lead to maternal flares.

Search terms used are as follows: pemphigoid gestationis, herpes gestationis, autoimmune in pregnancy.

## 2. Patient Case Presentation 

A 36-year-old Caucasian G4P1 presented with pruritic papules on bilateral anterior thighs at 18 weeks of gestation. The patient is, otherwise, a healthy female with no past medical or surgical history. She was only taking prenatal vitamins and fish oil at time of her first presentation. Previous pregnancies resulted in a full term SVD without complications in the spring of 2012, a missed abortion in 2010 at 12 weeks, and a SAB at 8 weeks in the spring of 2013. She has no significant family history of dermatological conditions or autoimmune diseases. The patient is married, never smoked, drinks occasional wine while not pregnant, has no history of street drugs, has no drug allergies, and is not on a special diet.

**Figure 1:**
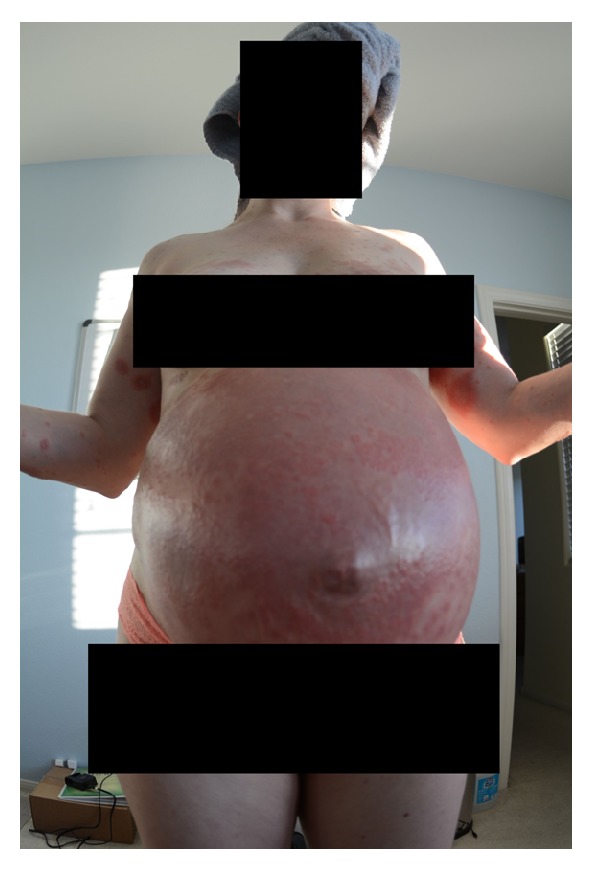


**Figure 2:**
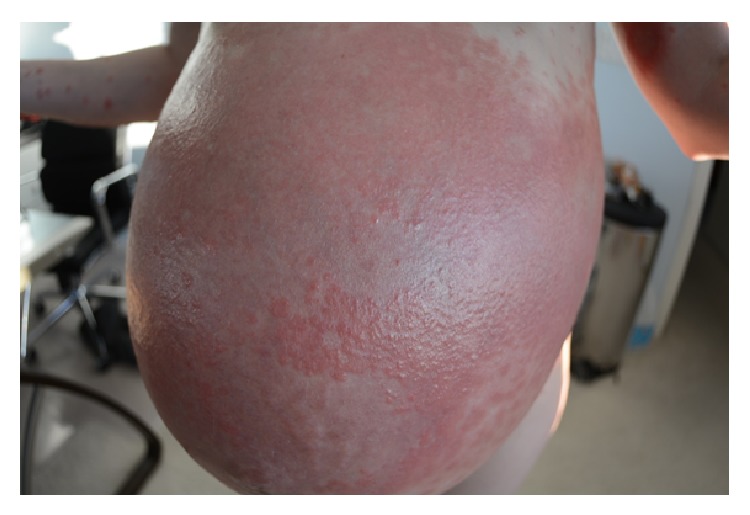


**Figure 3:**
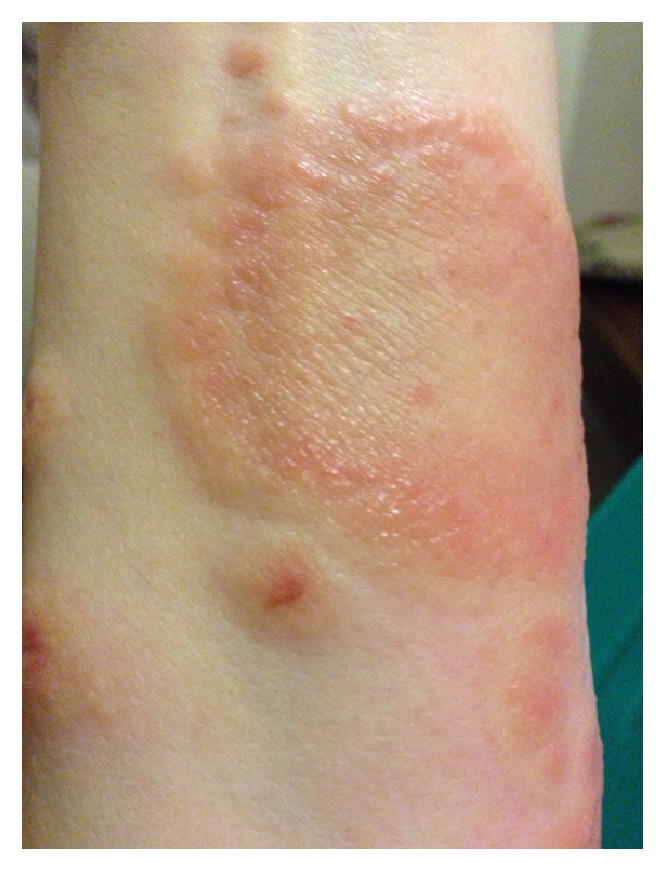


**Figure 4:**
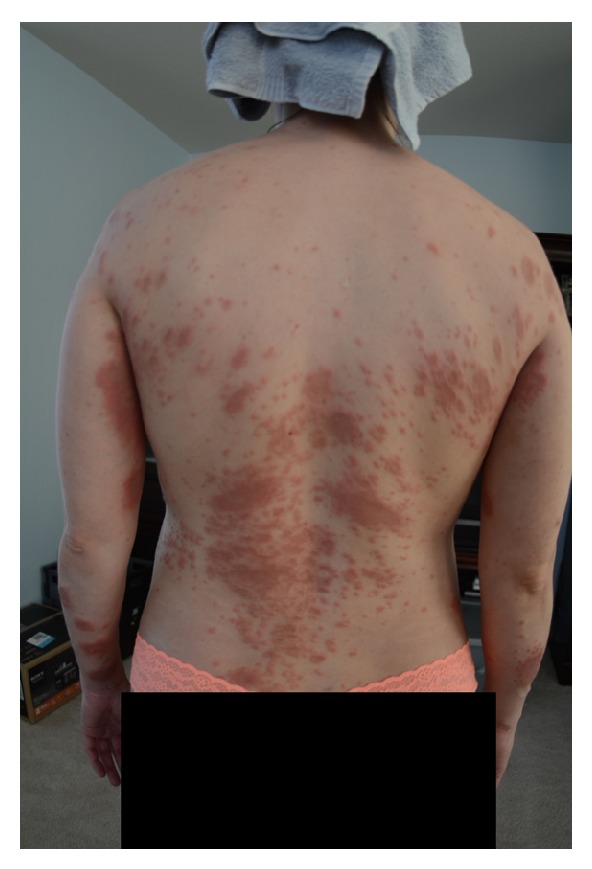


**Figure 5:**
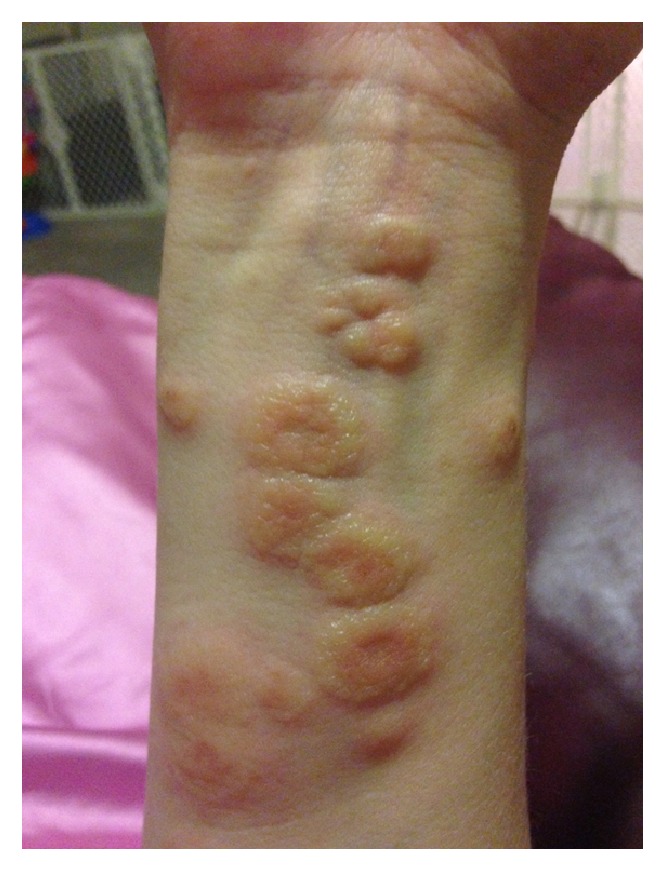


At 18 weeks of gestation, the patient presented with pruritic papules on bilateral anterior thighs. She was diagnosed with an atypical presentation of PUPPS and initially managed with triamcinolone cream BID, Claritin 10 mg QD, and Benadryl 25 mg QHS. These treatments somewhat improved the pruritus but the rash continued. At 28 weeks of gestation, the pruritic papules spread to cover the entirety of the legs, arms, abdomen, and back. By 32 weeks of gestation, the patient was no longer able to sleep or wear clothing due to the intense itching and irritation, despite attempting common home remedies for itching. Small vesicles started to appear in a circular ring on her left forearm surrounded by erythema with a fine scale and central clearing. Her abdomen was hot to touch and erythematous, with dependent edema under her umbilicus. She also presented with a yeast infection and fever at the time of her 31-week visit and was started on Diflucan 150 mg weekly, Clobetasol ointment BID, Keflex 750 mg BID, and a solu-medrol dose pack by her OB/GYN. At her 32-week visit, the patient's symptoms had not improved despite medication. She was admitted for 24 hour observation at 33 weeks and given two doses of betamethasone IM 24 hours apart. A reassuring nonstress test and biophysical profile were obtained during admission. Dermatology was consulted and confirmed the diagnosis of presumed PUPPS and prescribed Atarax 20 mg QHS for sleep, Zantac 150 mg QD, and Colace 100 mg BID. More vesicles appeared on the bilateral forearms and, by 34 weeks, she had some scattered bulla on her abdomen. Erythema, papules, and vesicles covered most of her body, to include palms, soles, chin, eyelids, back, abdomen, buttocks, mons pubis, labia, and all four extremities. The erythema and papules coalesced together until complex irregular plaques were formed. Acupuncture treatment provided minimal relief from the itching with no resolution of the rash. After dermatological evaluation, a biopsy was performed and the patient was started on prednisone 40 mg daily. By 35 weeks of gestation, the biopsy results were available which, when correlated with clinical presentation, was consistent with a diagnosis of pemphigoid gestationis. Histopathology showed marked interstitial, perivascular, and focal interface chronic dermatitis with mild spongiosis and subepidermal vesiculation. There were noted to be numerous superficial and middermal eosinophils, lymphocytes, mast cells, histiocytes, and a few scattered neutrophils. Direct immunofluorescence showed a linear pattern at the dermal-epidermal junction with C3. PAS test was negative. All serology was normal. The rash progressed to having vesicles along the outer edge of the plaques only, bulla resolved, and erythema was starting to fade into hyperpigmented patches covering the patient's body. She had an uncomplicated SVD of a healthy female infant at 37 weeks of gestation and was immediately tapered off steroid treatment by her OB/GYN to 20 mg prednisone PO QD. This resulted in a severe postpartum flare of her disease, with new lesions on her scalp, neck, and ears, as well as flaring of previous locations. Her care was then transferred to a rheumatologist and she was restarted on 60 mg of prednisone daily and tapered off weekly by 5 mg, until she was on 20 mg and then tapered by 1 mg a week. Each week of the taper the patient had some new small vesicles on her thighs, flanks, hands, and feet but overall showed improvement. A Mirena IUD was placed at her 6-week postpartum visit. At present, the hyperpigmentation seems to be fading without scaring and the pruritus is limited to the new lesions. Due to the many flares with tapering, it took 5 postpartum months to wean her off the steroids completely. Although the patient was experiencing some Cushing-like symptoms with prolonged steroid use, she chose to tolerate those over the multiple blisters. The patient has several striae that developed several weeks postpartum and she had a difficult time losing the weight gained during pregnancy, despite breastfeeding and exercise. At the time of this case report submission, she was 9 months postpartum and still having flares on her arms, legs, hands, and feet (see Figures 1, 2, 3, 4, and 5).

## 3. Discussion 

Pregnancy is a homeostatic state where selective suppression of the maternal immune system allows fetal tolerance. Fetal trophoblastic cells are typically devoid of MHC molecules, so the maternal immune system does not mount an immune response against the growing fetus [7]. However, in PG, it is thought that an aberrant expression of MHC class II antigens in the placenta triggers an autoimmune response as it invades the decidua, usually during the second trimester. This is an allogeneic response against Collagen XVII (BP180), a transmembrane hemidesmosomal 180 kDa protein, which is found in both the basement membrane zone (BMZ) of the skin and the amniotic epithelium of the placenta and umbilical cord [4]. IgG1 and IgG3 bind to the NC16A domain of the bullous pemphigoid antigen (BPAg2), activating the classical complement cascade leading to the characteristic C3 linear deposition at the dermal-epidermal junction. Complement leads to chemotaxis of eosinophils to the site of the AG-Ab complex on the BMZ, where they degranulate and damage the dermal-epidermal junction and cause blisters [5]. All PG patients have C3 deposits but only 50% had IgG by DIF. IgG4 is the subclass that can cross the placenta, so if the infant had gradually developed an immune response to the mother this explains why it would not show up until several months into the pregnancy and disappear shortly after the delivery [8].

There is a wide range of presentation and severity of PG as with most autoimmune diseases. Those pregnant women with preexisting autoimmune diseases have a variable course, with some improving during pregnancy (i.e., RA, MS, and thyroid dz) and some flaring (i.e., SLE) [9]. Pemphigoid gestationis is the only autoimmune disease known to be exclusively associated with pregnancy [2]. There is a combination of both endocrine and immune systems involved with PG. Patients at risk for PG are those with MHC class II HLA antigens DR3 (61–85%), DR4 (52%), or both DR3 and DR4 (43–45%) [10]. PG is also effected by estrogen and progesterone levels, thus the flares are often seen with menses and OCP use. Progesterone, which is elevated in the last few weeks of pregnancy, depresses antibody production, while estrogen enhances antibody production. This could explain why symptoms may improve just before delivery and worsen immediately postpartum when progesterone drops [11]. This hormone balance may play a part in postpartum management with birth control; however, there were no birth control suggestions in the literature. It has also been noted that prolactin has a stimulatory effect on antibody production but, overall, it suppresses the immune system. It has been suggested that breastfeeding may reduce postpartum duration of disease by increasing the immune suppression effects of prolactin [2].

Our case had very good examples of targetoid lesions confused with tinea at time of presentation. Typical presentation of PG is pruritus followed by the appearance of erythematous urticarial papules and plaques that may become target-like or polycyclic [4]. The lesions then form tense vesicles and bullae approximately 4 weeks after plaques and papules form. The vesicles and bullae then burst, leaving painful erosions and residual hyperpigmentation [6]. In most women (50–90%), distribution starts periumbilically and spreads to the rest of the body, including the palms and soles [12]. In one study, the thighs were the most common individual site [8] as was in our case. PG usually spares the face and mucous membranes and is found in <10% of PG patients; [13] however, our patient had it in both of these areas. The mean onset is between 21 and 28 weeks of gestation and rarely presents in the first trimester or postpartum. Greater than 75% of women have a postpartum flare with mean symptom duration of 28 weeks after birth [1]. Flares have also been noted with subsequent pregnancies, menses, and OCP use. Some reports found that up to half of the patients have recurrent flares between 3 months and 2 years after delivery [8]. General symptoms also reported include exhaustion, fevers, chills, and psychic distress due to severe pruritus [6]. The patient in our case study reported all of these at some point in her disease process.

Chronic PG lasting more than 6 months after pregnancy is rare, with only a few case reports noted. Patients with chronic PG were older and multigravid and had a history of PG during previous pregnancies. They also had widespread cutaneous eruption and mucosal involvement as well as IgG deposits on DIF and circulating serum antibodies IIF. No patient with only C3 deposits on DIF or IIF ended up having chronic PG [13]. A diagnosis of PG carries an increased risk for developing other autoimmune diseases later in life. This is most likely due to presence of HLA-DR3 and DR4, independently associated with autoimmune diseases. The most common reported comorbidity is Graves, at just over 10% [14].

Diagnosis of PG is made clinically and with direct IF as the gold standard. Histopathology will show eosinophilic spongiosis, subepidermal blister, dermal infiltrate of eosinophils, and lymphocytes. DIF will show linear deposition of C3 and IgG along BMZ. Optional serum studies include IIF (circulating IgG against BMZ), ELISA (circulating IgG against collagen XVII), and HLA profile of DR3 and 4 [5]. ELISA allows a serum diagnosis with 93% sensitivity and 100% specificity, which is better than IIF of 74% sensitivity and 80% specificity. However, with IIF and ELISA together, 100% of PG patients can be identified. It is currently recommended that DIF, IIF, and ELISA should be performed on patients suspected to have PG. Of note is that, unlike DIF, ELISA is quantitative, and therefore can be used diagnostically as well as using serum levels to correlate with disease severity as well as response to disease management [15].

Topical steroids may be used in mild cases of PG, but most patients require treatment with systemic steroids. Most women are started on prednisolone 20–40 mg daily for 1 to 2 weeks. If no new lesions reemerge for 3 days, it should be tapered off but increased again shortly after delivery [4]. Most patients can be tapered off permanently weeks after delivery; however, some have a refractory course for months to years. Those patients may benefit from immunosuppressive and anti-inflammatory agents, such as cyclophosphamide, azathioprine, rituximab, dapsone, pyridoxine, or methotrexate. Other treatments consist of IVIG, doxycycline/minocycline, plasmapheresis, or chemical oophorectomy with goserelin. Other helpful treatments include antihistamines, cool compresses, and drainage of larger blisters, without unroofing them, to avoid secondary infection. Vaseline gauze or silicone dressings might be helpful for larger lesions [16] (see Table 1).

The risk to the fetus is related to the slight alteration of the placental morphology that leads to relative placental insufficiency. This small change is enough to cause premature delivery and is small for gestational age fetuses in women with PG. Although the extent of disease and the amount of autoantibodies found do not correlate with adverse pregnancy outcomes, early onset of disease does. Earlier onset of PG exposes the fetus to a longer period of placental failure leading to LBW and preterm labor [17]. Neonatal PG occurs in only 10% of infants born to PG mothers. Symptoms include a range of dermatological lesions from erythema and papules to extensive bullae and denuded ulcers, as seen in Bedocs' case study [18]. Screening for adrenal insufficiency due to steroid use should be done if the mother takes >60 mg daily for an extended period of time. Systemic corticosteroid treatment does not negatively affect pregnancy outcome, thus justifying its use in PG pregnant women. It theoretically may even improve pregnancy outcomes by reducing placental inflammation [17]. There is no increased risk for spontaneous abortions or stillbirths in PG patients [19]. In our case, the infant was born 3 weeks early but had no complications.

It is important to keep a wide differential when cases present in an unusual manner. Our patient was initially diagnosed with PUPPS despite an irregular presentation with intense itching. Further, complicating matters, the targetoid lesions of PG may appear to be tinea. Direct IF is the gold standard for diagnosing PG. This case also demonstrates that early treatment with systemic steroids can ease a patient's PG symptoms and that positive outcomes result from gradual tapering followed by an increase immediately after delivery. Permanent taper can occur weeks after delivery. Future studies should target the possible relationship between birth control methods (e.g., IUD versus OCP) and flares in PG patients.

## Figures and Tables

**Table 1:** Differential diagnosis for a pruritic rash in pregnancy.

Pruritic urticarial papules and plaques of pregnancy (PUPPS) or polymorphic eruption of pregnancy (PEP) in the UK	Most common, usually presenting in the last trimester in the striae and sparing the umbilicus (Cobo et al., 2009 [5])

Bullous pemphigoid (BP)	Usually in elderly, starting on thighs, associated with HLA-DQ3 (Cobo et al., 2009 [5])

Dermatitis herpetiformis	Usually on extensor surfaces, associated with HLA-DQ2, DIF: IgA deposits (Lipozenčić et al., 2012 [6])

Erythema multiforme	Target lesions, usually after infection or drug, DIF: IgM deposits (Lipozenčić et al., 2012 [6])

Cicatricial pemphigoid	Involving mucous membranes

Linear IgA dermatosis	Associated with drug use, commonly vancomycin, DIF: linear IgA deposits

Acute urticaria	Wheal, flare; short duration

Allergic contact dermatitis	Typical linear, Rhus reaction

Papular dermatitis of pregnancy Or prurigo gestationis of Besnier	Usually on proximal limbs, DIF: linear IgM deposits

Pruritic folliculitis of pregnancy	Folliculitis and the DIF being negative

Scabies	Distribution: webs and belt lines and look for mites
